# Gut microbiome variations in *Rhinopithecus roxellanae* caused by changes in the environment

**DOI:** 10.1186/s12864-023-09142-6

**Published:** 2023-02-03

**Authors:** Gang Zhao, Mingpu Qi, Qiankun Wang, Changmin Hu, Xiang Li, Yingyu Chen, Jingyuan Yang, Huiliang Yu, Huanchun Chen, Aizhen Guo

**Affiliations:** 1 State Key Laboratory of Agricultural Microbiology, Wuhan, 430070 Hubei China; 2 College of Veterinary Medicine, Huazhong Agricultural University, Wuhan, 430070 Hubei China; 3 Hubei Hongshan Laboratory, Hubei International Scientific and Technological Cooperation Base of Veterinary Epidemiology, The Cooperative Innovation Center for Sustainable Pig Production, Huazhong Agricultural University, Wuhan, 430070 Hubei China; 4 Shennongjia Science & Technology Innovation Center, Huazhong Agricultural University, Wuhan, 430070 China; 5 National Professional Laboratory for Animal Tuberculosis (Wuhan), Key Laboratory of Development of Veterinary Diagnostic Products, Ministry of Agriculture and Rural Affairs, Huazhong Agricultural University, Wuhan, 430070 Hubei China; 6 Hubei Key Laboratory of Conservation Biology of Shennongjia Golden Monkey (Shennongjia National Park Administration), Shennongjia Forest Ecosystem Research Station, Shennongjia, 442411 China

**Keywords:** Gut microbiome, *Rhinopithecus roxellanae*, Metagenomics, Adaptation, Antibiotic resistant genes, *Bacteroidetes*/*Firmicutes* ratio

## Abstract

**Background:**

The snub-nosed monkey (*Rhinopithecus roxellanae*) is an endangered animal species mainly distributed in China and needs to be protected. Gut microbiome is an important determinant of animal health and population survival as it affects the adaptation of the animals to different foods and environments under kinetic changes of intrinsic and extrinsic factors. Therefore, this study aimed to elucidate gut fecal microbiome profiles of snub-nosed monkeys affected by several extrinsic and intrinsic factors, including raising patterns (captive vs. wild), age, sex, and diarrheal status to provide a reference for making protection strategies.

**Results:**

The 16S rRNA gene sequencing was firstly used to pre-check clustering of 38 fecal samples from the monkeys including 30 wild and 8 captive (5 healthy and 3 diarrheal) from three Regions of Shennongjia Nature Reserve, Hubei Province, China. Then the 24 samples with high-quality DNA from 18 wild and 6 captive (4 healthy and 2 diarrheal) monkeys were subjected to shotgun metagenomic sequencing to characterize bacterial gut microbial communities. We discovered that the raising pattern (captive and wild) rather than age and sex was the predominant factor attributed to gut microbiome structure and proportionality. Wild monkeys had significantly higher bacterial diversity and lower *Bacteroidetes/Firmicutes* ratios than captive animals. Moreover, the gut microbiomes in wild healthy monkeys were enriched for the genes involved in fatty acid production, while in captive animals, genes were enriched for vitamin biosynthesis and metabolism and amino acid biosynthesis from carbohydrate intermediates. Additionally, a total of 37 antibiotic resistant genes (ARG) types were detected. Unlike the microbiome diversity, the captive monkeys have a higher diversity of ARG than the wild animals.

**Conclusion:**

Taken together, we highlight the importance of self-reprogramed metabolism in the snub-nosed monkey gut microbiome to help captive and wild monkeys adapt to different intrinsic and extrinsic environmental change.

**Supplementary Information:**

The online version contains supplementary material available at 10.1186/s12864-023-09142-6.

## Introduction

Globally, the snub-nosed monkey (*Rhinopithecus roxellanae*) is an endangered Colobinae species, distributed only in China, Myanmar, and Vietnam, and thereby listed on the Red List of endangered species from the International Union for Conservation of Nature [1]. In China, these animals belong to the first class in national list of protected species which comprises mostly endangered animal species needed to provide government and non-government levels of measures for protection of their survival [2]. Currently, approximately 25,000 snub-nosed monkeys are distributed in Hubei, Sichuan, Shanxi, and Gansu provinces, China [3–5], and roughly 1,471 live in Shennongjia Nature Reserve, Hubei Province. In terms of accelerating ecological degradation worldwide, the pressure is mounting to protect this species in different regions and countries.

Food is critical to the survival and health of any animal population and food metabolism is associated with the gut microbiome, which is composed of a dynamic balance of trillions of microorganisms and provides specific services to digest certain foods [6, 7]. This complex system has an important role in maintaining several critical physiological activities such as metabolism and immune responses, or disease development [5, 8]. It is generally considered that the gut microbiome is structured by host dietary niches. However, many other factors might influence gut microbiome plasticity including both extrinsic environmental factors such as food resources, and raising patterns, and intrinsic factors such as species, age, sex, gut morphology, and health status [7, 9–11]. Ultimately, both host phylogeny and gut microbiome co-diversify to respond to physiological or environmental change. Therefore, to characterize the gut microbiomes of important species has become a hot area to elucidate the mechanisms of animal adaptation to environment and defense against infectious and non-infectious diseases.

Similar findings are obtained in research on the gut microbiomes in nonhuman primate (NHP) populations. Amato et al.found that the composition and function of gut microbiomes of NHP species were affected by gut morphological specializations much stronger than the dietary niche such as folivores or non-folivores [12]. Additionally, habitat degradation and disturbance significantly decreased gut microbial diversity in NHPs [13–15]. However, in Uganda, one study identified no association between gut microbial diversity and habitat degradation [16]. When compared with wild animals, gut microbiome α-diversity decreased in different captive NHPs [17, 18]. Furthermore, NHP captivity under different diet conditions generated significant differences in gut microbial composition [19]. Therefore, there are many unclear mechanisms to be clarified which contribute to gut microbiome structure and function.

Antibiotic resistance transfer is a hot area in human and veterinary medicine. The antibiotic resistance genes (ARG) can be transferred from one bacterium to others possibly inducing new antibiotic resistance. The human and animal gut microbiomes are reservoirs of ARG [20, 21]. With the advances in high-throughput sequencing technology and metagenomic analysis, the antibiotic resistomes can be extensively investigated. The related studies have uncovered high diversity and abundance of ARG in the human and animal gut microbiomes [21–23]. For example, a total of 1,093 antibiotic resistance genes were identified in human gut microbiomes varied between Chinese and European populations [23]. Therefore, antibiotic resistomes contribute to the diversity of gut microbiomes. In addition, an in-depth study of resistomes should help understand the way by which antibiotic resistance genes spread among livestock, environments, and human microbiomes.

*R. roxellanae* usually inhabits mountainous regions at elevations between 1500 and 3500 m above sea level. In the wild, monkeys can eat a wide range of food such as leaves, seeds, and bark, while in captivity, the animals are usually fed with very limited food kinds like fruits (apples, oranges, etc.) and root vegetables (such as carrots) [5, 24]. So far, the *R. roxellanae* gut microbiome has been preliminarily studied and showed that age [25] and living patterns (captive/wild) [5] affected the relative abundance of gut microbial species. Since the gut microbiome plays an important role in host metabolism and immunity and more than 400 individuals of *R. roxellanae* are being raised in captivity [26], it is of significance to study further the difference in gut microbiomes between the animals in captivity and wild to establish the stool microbial communities as biological markers for the health-status of this endangered NHP based on patterns in taxonomic abundance, proportionality, and richness. In addition, ARG of gut microbiome in *R. roxellanae* has not been extensively studied yet. Revealing ARG of gut microbiomes would help predict susceptible drugs for the monkeys when the animals are in need of veterinary medication and understand how antibiotic resistance genes circulate among this endangered species, surrounding livestock, and humans. In current study, 38 *R. roxellanae* fecal samples from Shennongjia Nature Reserve, Hubei province, China were pre-evaluated by 16S rRNA gene sequencing and taxonomic and functional gut microbiome level differences were further investigated in 24 monkeys using a metagenomic sequencing approach. Additionally, ARG distribution was investigated. Our results would help improve surveillance program and protection strategies for this endangered species.

## Materials and methods

### Animals, sample collection, and DNA extraction

Approximately, a total of 85 *R. roxellanae* were available for research in Shennongjia Nature Reserve. The monkeys were distributed across three regions: Region I: there were about 60 free-ranging monkeys lived in the Dalongtan area, which were composed of four one-male units (OMU) and one all male unit (AMU); Region II: Shennongjia snub-nosed monkey breeding base where 15 monkeys were kept and randomly divided into three OMUs by animal keepers; each unit was independently caged in one steel house; and Region III: Xiaolongtan area is the place where the injured monkeys rescued from different areas of Shennongjia Reserve were cared; 10 monkeys in a cage were taken as one unit. In total, we collected fresh fecal samples from 38 monkeys including 30 wild monkeys from Regions I and eight captive monkeys from II (six, three healthy and three diarrheal) and III (two, one healthy and one diarrheal monkeys) respectively in July 2012. Monkey age, raising patterns, sex, and health status were recorded (additional file 1). The monkey age was determined by their coat color, body length and wart-like growth at the corners of the mouth as described previously [27]. Meanwhile, the health status of the monkeys was clinically determined by observing their bright coats, movement (lively, powerful), good appetite, fecal shape, and color.

All the wild and captive monkeys were fed three times per day (10:00–11:00, 14:00–15:00, and 18:00–19:00). The wild monkeys from region I usually came quickly from the wild to the trees in the feeding place at the fixed time after hearing the call of the staff. Then they jumped down from the trees to get the food and ate it. After feeding, they immediately went back to the mountain for their free-living in the wild and could get more food in the forest. Meanwhile, the monkeys in regions II and III were kept and fed in their cages at similar timepoints.

Fecal samples were collected from the monkeys by using the protocol described previously [2]. Briefly, fecal samples were collected during one week by two researchers before feeding time in the morning (10:00–11:00) and afternoon (14:00–15:00), when the monkeys had already gone down the mountain, gathered in the trees at the feeding place and waited for their food. Each researcher was responsible for one monkey unit per time. Fresh fecal samples were immediately collected into sterile tubes. For the diarrheal fecal samples, sterile cotton swabs were used and dipped the feces in the center carefully for several times to collect feces as many as possible, and stored into sterile tubes as well. Samples were stored at -80 °C at Shennongjia Reserve and transported to our laboratory on dry ice and stored at -80 °C until DNA extraction. Microbial DNA from fecal samples was extracted using QIAamp DNA stool mini kits (Qiagen, CA, USA) following standard protocols.

### The 16S rRNA gene sequencing and data analysis

DNA quantity and quality were measured using a Nanodrop 2000 spectrophotometer (Nanodrop Technologies, DE, USA) and agarose gel electrophoresis, respectively. The 16S rRNA gene V4 region in DNA was amplified using specific primers (515F and 806R), which were incorporated with barcodes [28]. Polymerase chain reaction (PCR) was performed using a Phusion® High-Fidelity PCR Master Mix (New England Biolabs, MA, USA). Amplicons were extracted from 2% agarose gels and purified using a Qiagen Gel Extraction Kit (Qiagen, Germany). Sequencing libraries were generated using a TruSeq® DNA PCRFree Sample Preparation Kit (Illumina, CA, USA) following manufacturer’s protocols, and index codes were added. Library quality was assessed on the Qubit@ 2.0 Fluorometer (Thermo Scientific, MA, USA) and Agilent Bioanalyzer 2100 system. The library was then sequenced on an Illumina Miseq platform and 250 bp paired-end reads generated.

After assigning paired-end reads to samples based on unique barcodes, reads were merged using FLASH (Version 1.2.7) [29], and high quality tags obtained using a QIIME (Version 1.7.0) [30] quality control process. After detecting chimeric sequences using the UCHIME algorithm [31], effective tags were finally generated for analysis.

To determine tag taxonomic annotation, tags were clustered into operational taxonomic units (OTUs) using de novo OTU clustering method in Uparse [32] with a 97% sequence identity, the highest frequency sequence in OTUs was selected as the representative sequence, and taxonomic data were then assigned to each representative sequence in Greengenes [33] database using the Ribosomal Database Project (RDP) classifier. OUT abundance information of each sample was rarified using a standard of sequence number corresponding to the sample with the least sequences. Alpha diversity was applied to analyze the complexity of species diversity for a sample through Shannon. Meanwhile, Beta diversity analysis was used to evaluate differences of samples in species complexity. Beta diversity on both weighted and unweighted Unifrac was calculated. All these indices were calculated with QIIME (Version 1.7.0) and displayed with R software (Version 2.15.3).

### Shotgun metagenomics sequencing and data analysis

The high-quality DNA samples without degradation from each group were selected including 18 wild monkeys from Region I and six captive monkeys (three healthy and two diarrheal from Region II and one healthy from Region III) for shotgun metagenomics sequencing. Briefly, samples were paired-end sequenced on the Illumina platform (insert size = 300 base pairs (bp), read length = 100 bp) at Novogene Bioinformatics Technology Co., Ltd (Tianjin, China). After quality control, high quality read assembly was executed in SOAPdenovo v. 2.04 [34] (parameters: -R -d 1 -D 1 –F). Genes (minimum coding length = 34 amino acids (AAs)) were predicted on scaftigs (i.e., continuous sequences within scaffolds), with genes > 300 bp predicted using MetaGeneMark [35]. Then, a nonredundant gene catalog was constructed in CD-HIT (Version 4.5.8, parameters: -G 0 -aS 0.9 -g 1 -d 0 -c 0.95) [36] using a sequence identity cut-off = 0.95 and a minimum coverage cut-off = 0.9 for shorter sequences.

To assess gene abundance, reads were realigned to the gene catalog with SOAP2 [37] using parameters: -m 200—× 400 -s 95. Gene abundance was calculated by counting the number of reads aligned to the gene when normalized by gene length. Genes were aligned to the integrated Non-Redundant Protein Sequence Database (NR), Kyoto Encyclopedia of Genes and Genomes (KEGG) [38] (Release 73.1, with animal and plant genes removed), eggNOG (Version 4.1) [39] and Carbohydrate-Active enZYmes (CAZy) databases (Version 2014.10.20) [40] using DIAMOND (Version 0.7.9.58, default parameter except that -k 50 –sensitive -e 0.00001) [41]. Significant gene matches, which were defined by e-values ≤ 10 × the smallest e-value of the top hit chosen to take the LCA algorithm, were retained to distinguish taxonomic groups. The taxonomical level of each gene was determined by the lowest common ancestor (LCA)-based algorithm in MEGAN [42]. For functional analysis, proteins were assigned to the KEGG Orthology (KO) by the highest scoring annotated hit(s) containing at least one HSP scoring over 60 bits [43]. Feature abundance was calculated by summing gene abundance annotated to the same feature.

Sequencing reads were aligned to the Comprehensive Antibiotic Resistance Database (CARD) to identify ARG with an e-value cutoff of 1e-5 by using BLAST. Sequence identity was ≥ 90% and alignment length was ≥ 30 AAs. Gene level data were used to calculate ARG richness, while normalized data, aggregated from gene level outputs to group and class levels, were used to generate heat maps.

### Statistical analysis

Genera abundance was defined by MetaStats (Version 2009.04.14) as significantly different when the *p* value was ≤ 0.05 and the *q* value was ≤ 0.05 [44]. Abundance differences between KO, corresponding enzyme (EC), and carbohydrate-active enzymes families were tested using Wilcoxon rank sum tests, and *p* values were corrected for multiple testing using the Benjamin & Hochberg method. To identify differential species between diarrheal and healthy monkeys, *t*-test was conducted for each species from species level profiles. Species with a *p* value < 0.05 was identified as significantly differential species.

Spearman’s correlations were calculated based on differential genera and ECs profiles, and *p* values were corrected for multiple testing using the Holm method in R (Version 2.15.3, psychpackage).

Based on genus profiles, the Shannon index was calculated in QIIME (Version 1.7.0) to identify within-sample (α) diversity. Principal component analysis (PCA) was performed using the FactoMineR package in R software (Version 2.15.3). Principal coordinate analysis (PCoA) was performed and displayed in ade4, cluster, fpc, and clusterSim packages in R software (Version 2.15.3).

## Results

### Gut microbial structures in *R. roxellanae*

**Fig. 1:**
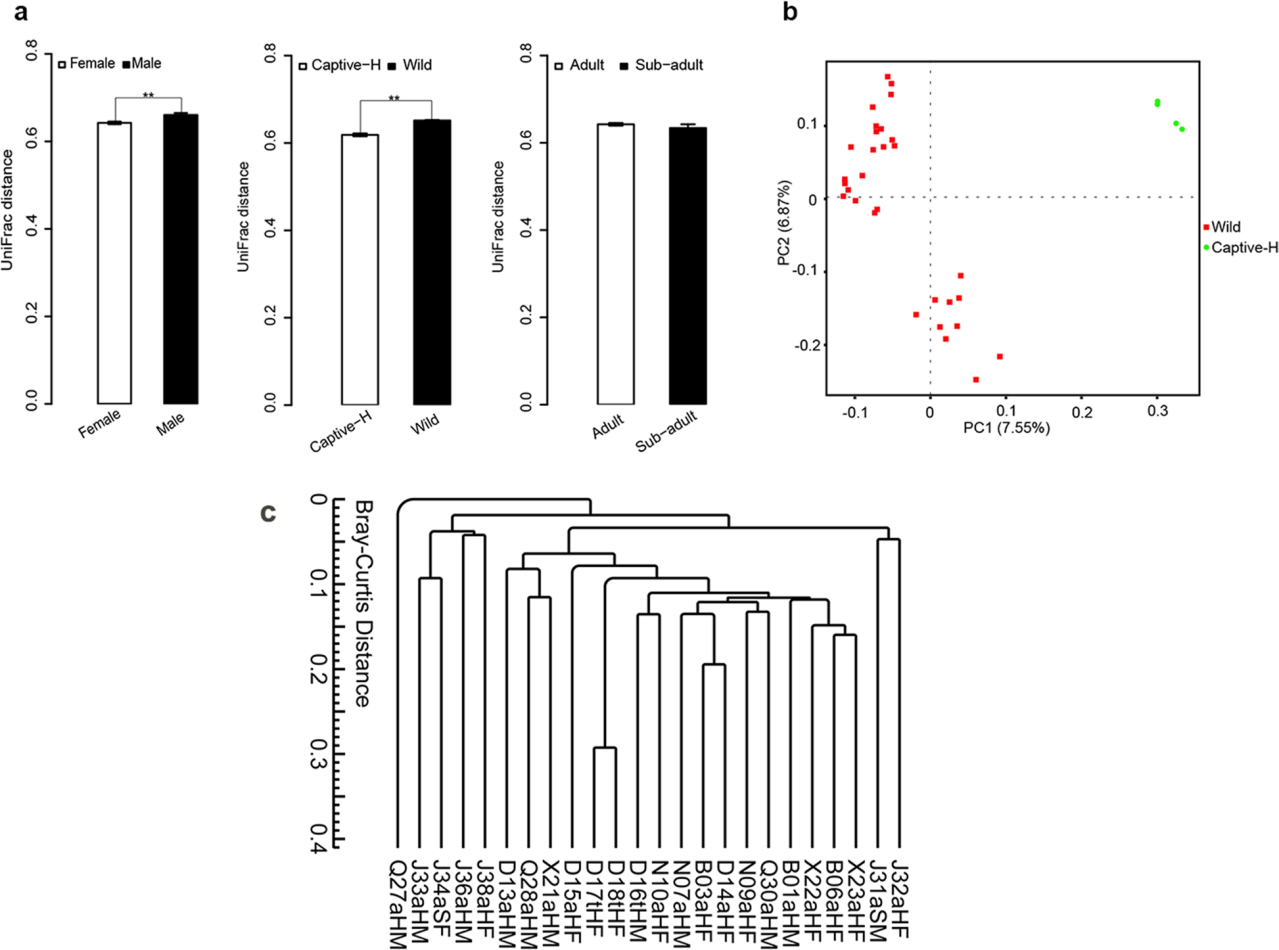
Difference in the fecal microbial communities of *R. roxellanae*. **a** The similarity of fecal microbiomes measured by UniFrac distance considering sex, raising patterns, and age. Results are derived from bacterial 16 s V4 rRNA data sets. **, *p* < 0.01 (Kruskal–Wallis test). **b** PCoA based on the Unweighted UniFrac distance from the genera profiles of the captive healthy monkeys (Captive-H)and wild monkeys (Wild). **c** The dendrogram based on each sample was developed demonstrating all 6 captive healthy monkeys were clustered together, while the wild monkeys were better clustered into another group compared PCoA

After annotating 16S rRNA gene sequencing data of the 38 fecal samples, 54,433 OUTs were generated, and 20,680 OTUs were left for further analysis after data normalization (additional file 2). We used UniFrac distance to measure microbial similarity levels in samples (additional file 2). Our data indicated that both the raising patterns (wild vs. captive, *p* = 0.0024) and sex (male vs. female, *p* = 0.0041) were major forces driving microbial community variations among the selected factors (Fig. 1a). However, PCoA analysis based on the UniFrac distance revealed that only the raising patterns (wild vs. captive) rather than sex or age presented the separated clusters of gut microbiomes (Fig. 1b, and c) (Fig. S1, additional file 3, and additional file 4). Compared to PCoA analysis, the dendrogram better demonstrated clustering of most wild monkeys except one monkey Q27aHM (Fig. 1c).

**Fig. 2:**
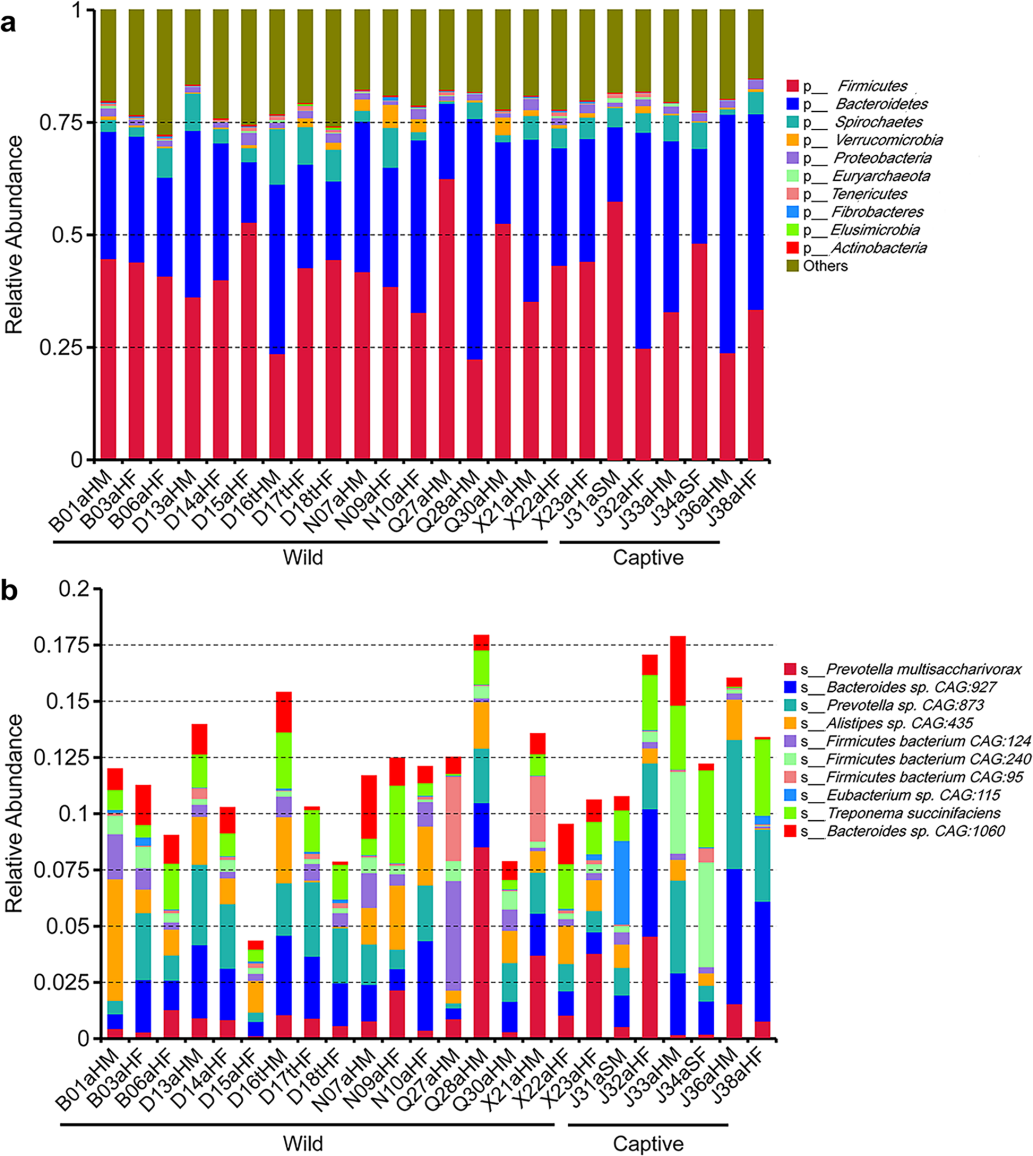
Community constituents of gut microbiomes among *R. roxellanae* based on shotgun sequencing of fecal DNA from a subset of 24 fecal samples. **a** The top 10 phylum of the *R. roxellanae* gut microbiome. Each column represents the gut microbial community from a single monkey. Phylum *Firmicutes* and *Bacteroidetes* made up the majority of phyla in each monkey. **b** The top 10 species of the *R. roxellanae* gut microbiome. Captive: captive monkeys; Wild: wild monkeys

For the shotgun metagenomic sequencing, a total of 139,935 Mb data were generated, with an average of 5,830 Mb data/sample. Also, 4,152,852 genes were predicted using MetaGeneMark. All data-related statistical information from sequencing, contig assembly, and predicted open reading frames was listed (additional file 5). The top 10 bacteria of gut microbiomes at the phylum level were shown (Fig. 2a); *Firmicutes* and *Bacteroidetes* were the most dominant bacteria in all 24 samples. Almost all the top 10 bacteria in the wild and captive monkey microbiomes were: *Prevotella multisaccharivorax*, *Bacteroides sp. CAG:927*, *Prevotella sp. CAG: 873*, *Alistipes sp. CAG: 435*, *Firmicutes bacterium CAG:124*, *Firmicutes bacterium CAG:240*, *Firmicutes bacterium CAG:95*, *Eubacterium sp. CAG:115*, *Treponema succinifaciens*, and *Bacteroides sp. CAG:1060* (Fig. 2b).

### Fecal microbial community differences between captive and wild monkeys

**Fig. 3:**
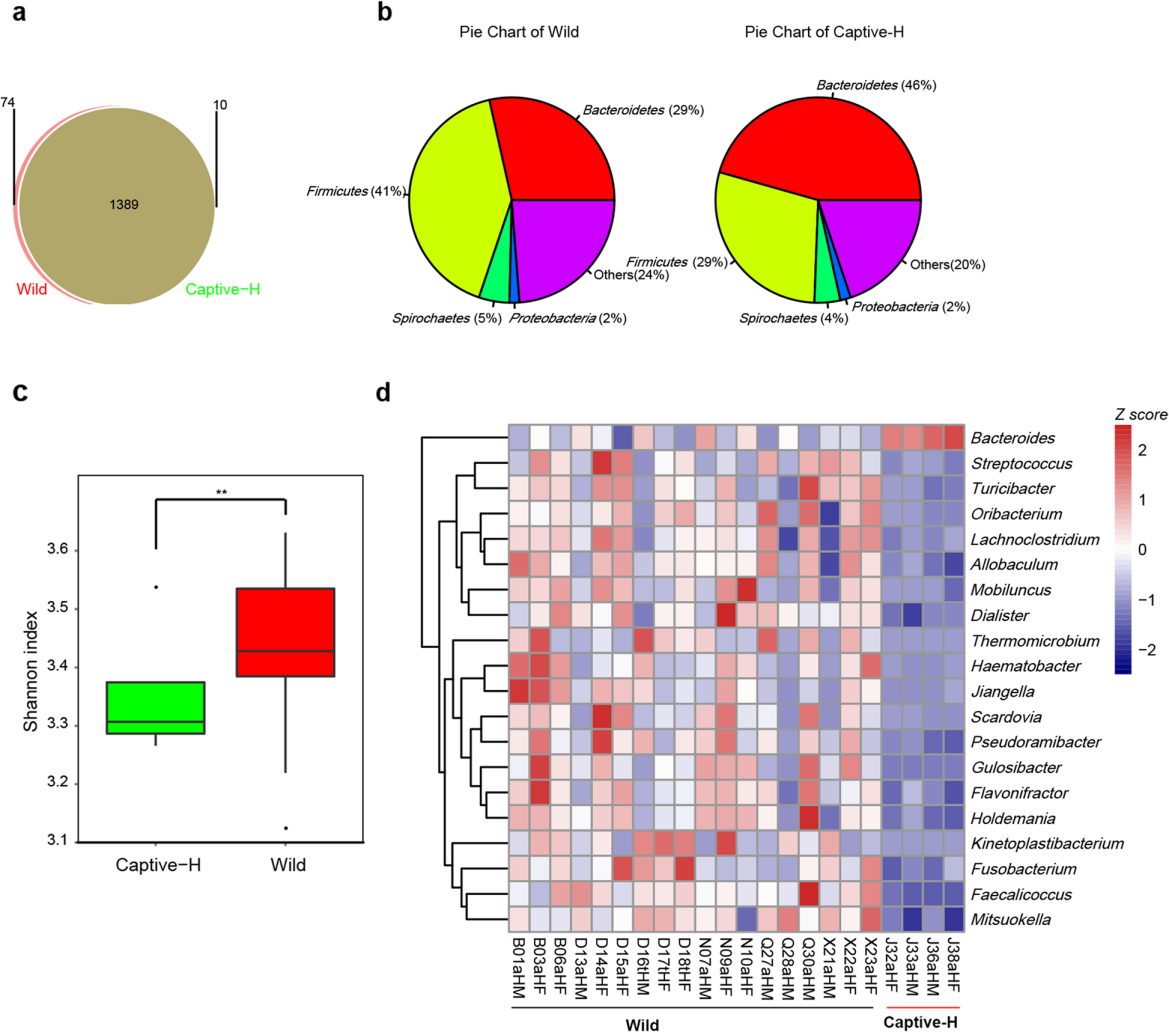
Microbial community profiles of captive healthy monkeys (Captive-H) and wild monkeys (Wild) based on shotgun sequencing. **a** The Venn diagram of the microbial composition of wild and captive healthy monkeys at the genus level. Wild monkeys have 74 unique genera, while captive healthy monkeys have 10 unique genera. **b** The pie chart of most dominant phylum in wild monkeys or captive healthy monkeys. **c** Comparison of the Shannon index among wild monkeys and captive healthy monkeys based on the genera profile. Wild monkeys represent higher bacterial diversity than captive healthy monkeys (*p* < 0.01). **d** The differential genera between captive healthy monkeys and wild monkeys measured by MetaStats (Version 2009.04.14, *p* value ≤ 0.05, *q* value ≤ 0.05)

After gene annotation using the NR database, taxonomic profiles at different levels were generated. From genera profiles, wild monkeys had 74 unique genera, while captive healthy monkeys had 10 (Fig. 3a). Pie charts were constructed and revealed apparent differences of fecal microbial communities between captive healthy and wild monkeys (Fig. 3b). Meanwhile α-diversity indices verified that the difference was statistically significant between Captive-H and Wild groups (*p* = 0.0013) (Fig. 3c). These findings indicated that bacterial gut microbiome diversity in wild monkeys was higher than captive monkeys. Additionally, based on genus level profiles (additional file 6), MetaStats software was used to identify significant differential genera between wild and captive healthy monkeys (*p* < 0.05 and *q* < 0.05). Heat map data showed that the dominant genera in fecal microbiomes of wild monkeys were *Faecalicoccus*, *Mitsuokella*, and *Fusobacterium*, while *Bacteroides* in captive monkeys (Fig. 3d) (additional file 7).

### Global metabolism features in monkey gut metagenomes

To assess gut microbiome gene functions in monkeys, we aligned gene catalogs to KEGG and eggNOG database. KEGG metabolic pathways provided a highly integrated overview of global metabolism (Fig. S2), indicating that the monkey gut microbiome was enriched in carbohydrate, AA, nucleotide, energy, vitamin, and lipid metabolism. The eggNOG database was also used to assess the cluster abundance of orthologous monkey groups, while COG (Clusters of Orthologous Groups) function annotation showed that function unknown, carbohydrate transport and metabolism, AA transport and metabolism, and energy production and conversion were enriched in fecal microbiomes (Fig. S3 and additional file 8).

After global metabolism analysis, we compared fecal microbial community gene functions between the wild and captive healthy monkeys, and revealed that wild monkeys had unique genes involved in carbohydrate metabolism, vitamin metabolism, and biosynthesis of other secondary metabolites pathways (Fig. S2, labeled with bold red lines). Meanwhile, captive monkeys had unique genes involved in amino acid metabolism pathways (Fig. S2, labeled with bold green lines).

### Metabolic differences between captive and wild monkeys

**Fig. 4:**
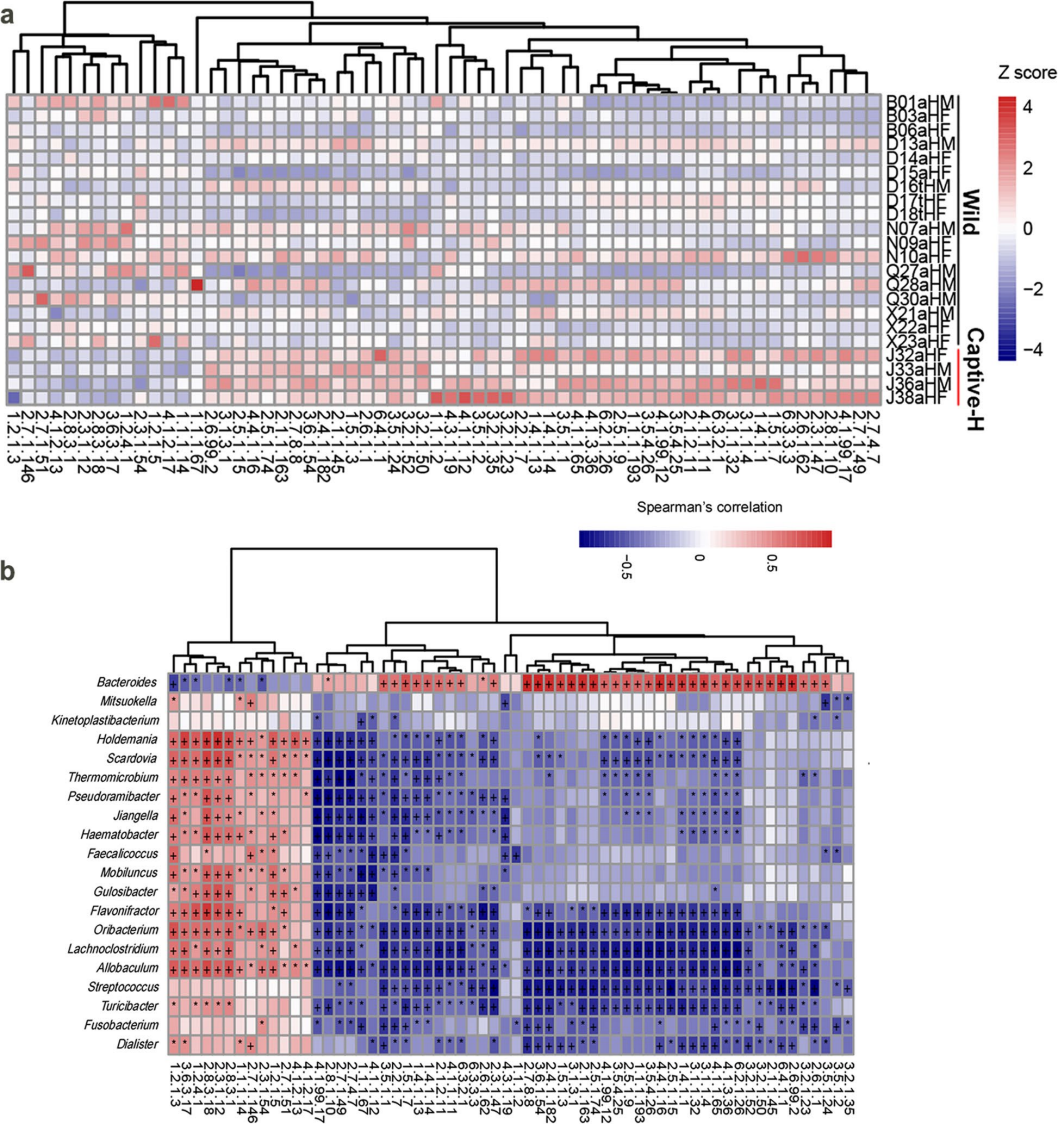
The differential functional genes between captive healthy monkeys (Captive-H) and wild monkeys (Wild). **a** Heatmap of the differential KEGG ECs between wild monkeys and captive healthy monkeys, which were involved in the carbohydrate, amino acid, and vitamins metabolism. The abundance profile was transformed into Z scores by subtracting the average abundance and dividing the standard deviation of all samples. **b** Spearman’s correlation between differential genera and differential ECs involved in the carbohydrate, amino acid, and vitamins metabolism. The color was scaled with the correlation coefficients. + , adjust *p* value < 0.01; *, adjust *p* value < 0.05

From KEGG EC profiles across 22 fecal samples (additional file 9), 367 ECs were identified at different proportions between 18 wild and 4 captive healthy monkeys. The wild monkeys had 149 ECs with higher abundance, while 218 with lower abundance when compared with captive healthy monkeys (*p* < 0.05, *q* < 0.05, additional file 10). After preliminary analysis, some ECs related to carbohydrate, AA, and vitamin metabolism were selected to illustrate differences in functional features (Fig. 4a). The wild monkey gut microbiomes were enriched in genes involved in acetate biosynthesis: ADP-specific phosphofructokinase (EC2.7.1.146) and 1,6-diphosphofructose aldolase (EC4.1.2.13) in glycolysis, mannitol 2-dehydrogenase (EC1.1.1.67) in mannitol utilization, and L-fuculokinase (EC2.7.1.51) and L-fuculose-phosphate aldolase (EC4.1.2.17) involved in fuculose degradation. Spearman’s correlation analysis was used to explore correlations between gut microbiomes and ECs. A higher relative abundance of acetate biosynthesis ECs in the fecal microbiome of wild monkeys correlated with higher representative numbers of *Erysipelotrichaceae*, *Lachnospiraceae*, *Scarddovia*, *Thermomicrobium*, *Pseudoramibacter*, *Jiangella*, *Haematobacter*, *Mobiluncus*, *Gulosibacter*, and *Flavonifractor* (Fig. 4b).

**Fig. 5:**
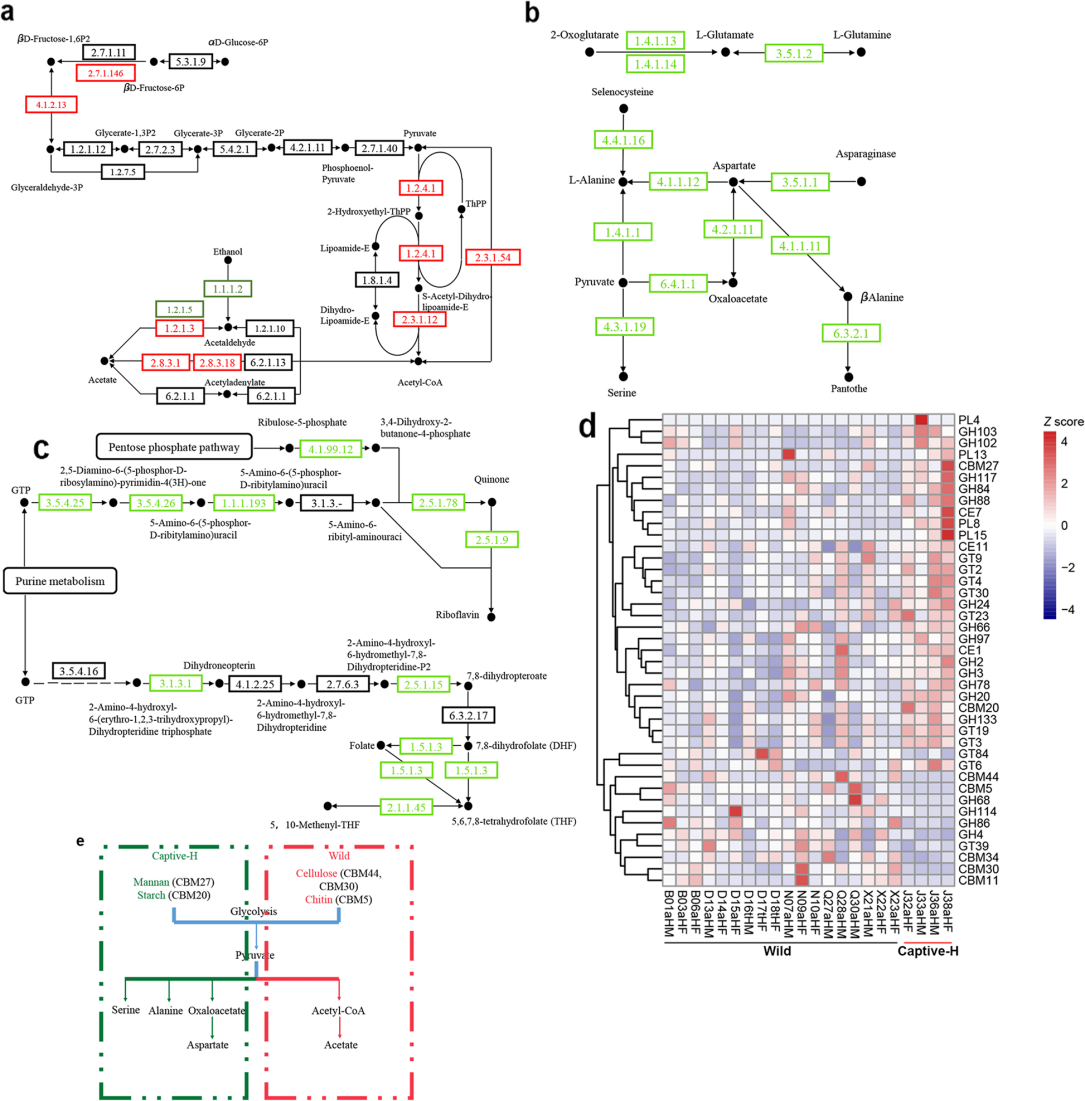
Differences in metabolism of sugar and vitamin synthesis between captive healthy monkeys and wild monkeys. Diagram of KEGG pathway for glycolysis (**a**) and pyruvate metabolism (**b**). The ECs colored in red and the ECs colored in green indicated a higher abundance of Wild monkeys and Captive healthy monkeys respectively. **c** Diagram of KEGG pathway for riboflavin and folate biosynthesis, ECs colored in green represent a higher abundance of captive healthy monkeys when compared with wild monkeys. The *p* values and *q* values for the ECs can be found in additional file 10. **d** The differential CAZy families between captive healthy monkeys and wild monkeys. The abundance profile was transformed into Z scores by subtracting the average abundance and dividing the standard deviation of all samples. **e** Diagram for difference in metabolism of sugar. The red box represented the tendency for wild monkeys to utilize the sugar in comparison with captive healthy monkeys (green box). The *p* values and *q* values for the differential ECs and CBMs can be found in additional file 10 and additional file 12 respectively

In contrast, the proportion of genes encoding enzymes involved in pantoate, thiamine, biotin, vitamin B6, and vitamin K biosynthesis were enriched in captive monkey microbiomes. Vitamin biosynthesis gene enrichment correlated with increased *Bacteroides* numbers (Fig. 4c). Additionally, captive monkeys had a higher abundance of ECs which catalyzed AA formation, such as serine, glutamate, alanine, and aspartate from carbohydrate intermediates such as pyruvate, oxaloacetate, and oxoglutarate, when compared with the wild monkeys (Fig. 5a and b). Also, the genes encoding EC2.7.8.8, EC3.1.1.32, EC3.1.1.4, and EC4.1.1.65 involved in glycerophospholipid metabolism were significantly more abundant in captive than in wild monkeys (additional file 10). The higher relative abundance of ECs involved in AA formation and glycerophospholipid metabolism correlated with higher *Bacteroides* numbers. Additionally, genes involved in vitamin biosynthesis pathways were significantly more abundant in captive than in wild monkey microbiomes (additional file 10). For example, captive monkey microbiomes had a higher abundance of ECs involved in riboflavin (riboflavin synthase, EC2.5.1.9) and folate (dihydrofolate reductase, EC1.5.1.3) biosynthesis than captive monkeys (Fig. 5c).

After gene annotation using the CAZy database, functional profiles were generated and cluster analysis indicated that carbohydrate-active enzymes in wild and captive monkeys were distributed in two different branches (Fig. S4). Furthermore, carbohydrate-active enzymes family abundance was calculated for comparison (additional file 11). Generally, the higher relative abundance of glycoside hydrolases (GHs) and glycosyl transferases (GTs) in captive healthy monkey microbiomes indicated a greater ability to use diet-derived sugar (Fig. 5d). For example, enriched GH families (GH66, GH97, GH2, GH3, GH133, GH78, and GH20) are monosaccharide or disaccharide hydrolyzing enzymes (http://www.cazy.org/). Furthermore, carbohydrate-binding module 27 (CBM27) and CBM20 enrichment was identified in captive healthy monkey microbiomes, while CBM44, CBM30, and CBM5 enrichment were identified in wild monkey microbiomes, and supported the notion of diet shifts (Fig. 5e) (additional file 12). CBM27 and CBM20 bound mannan and cyclodextrins respectively, while CBM44, CBM30, and CBM5 bound cellulose and chitin (http://www.cazy.org/). These data showed that the captive and wild monkeys used different carbohydrate sources.

Together, these findings suggested that gut microbiomes in wild monkeys expressed differential ECs and carbohydrate-active enzymes when compared with captive healthy monkeys, indicating different metabolic features arising from the living environment.

### Diarrhea alters gut microbiomes

**Fig. 6:**
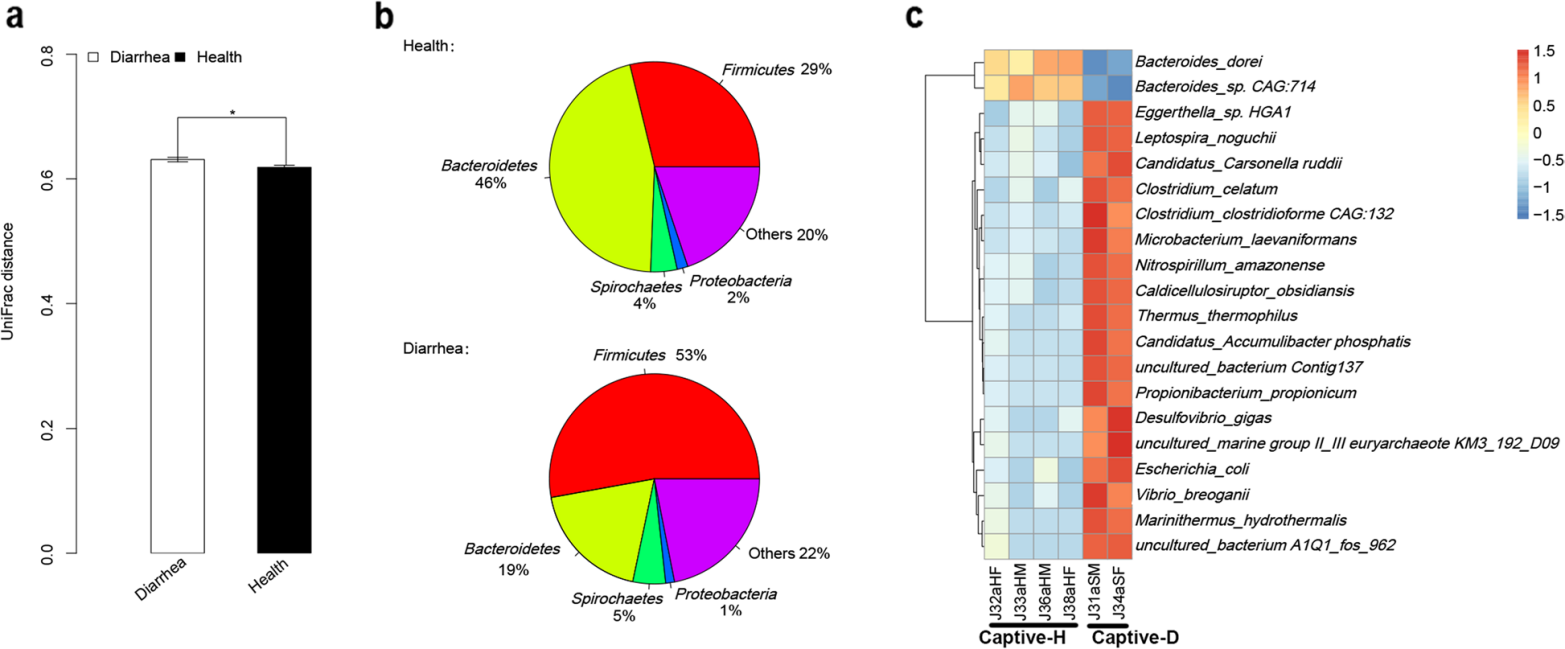
The difference in the fecal microbiomes between captive healthy monkeys (Captive-H) and diarrheal monkeys (Captive-D). **a** The similarity of fecal microbiota measured by UniFrac distance derived from bacterial 16 s V4 rRNA data sets. **b** The pie chart of most dominant phylum in healthy monkeys and diarrheal monkeys. **c** The heatmap of differential species (*p* < 0.001)

Differences in fecal microbiomes between healthy and diarrheal captive monkeys were identified by using UniFrac distance based on bacterial 16 s rRNA gene V4 data (*p* < 0.05) (Fig. 6a). Metagenomic sequencing further identified differences in microbial communities, while the pie charts of the most dominant phyla showed variations in microbial communities between diarrheal and healthy monkeys. Healthy monkeys had a lower abundance of *Firmicutes* (29% vs. 53%) and a higher abundance of *Bacteroides* (46% vs. 19%) when compared with diarrheal monkeys (Fig. 6b). Furthermore, the proportions of 20 fecal microbiome species differed significantly between healthy and diarrheal monkeys (*p* < 0.001). Of these, a higher proportion of *Bactroides_derei* and *Bacteroides_sp. CAG:714* were identified in the gut microbiomes of captive healthy monkeys, while *Eggerthella_sp. HGA1*, *Leptospira_noguchii*, *etc*had higher proportions in diarrheal monkeys (Fig. 6c).

### ARG in monkey gut microbiomes

**Fig. 7:**
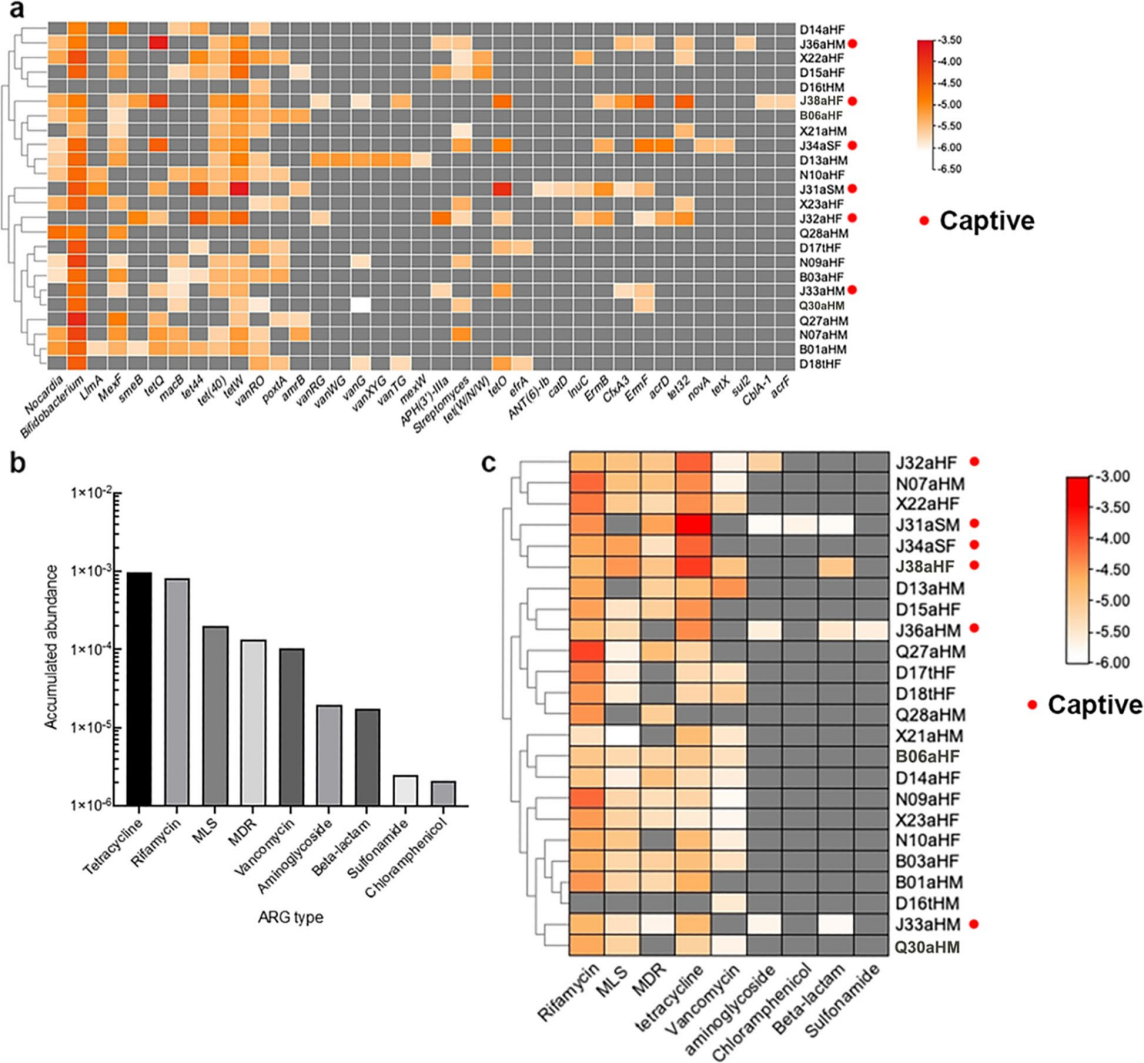
The antibiotic resistant genes (ARG) and the distribution of corresponding resistant drug classes in the gut microbiomes of *R. roxellanae*. **a** Broad spectrum profiles of the 37 ARGs in 24 samples. **b** Accumulated abundance of different ARG in 24 samples. **c** abundance of ARG in the 9 antibiotic classes in 24 samples. The abundance of ARG was transformed into log scores and illustrated in the heatmap by red to grey with the abundance of high to low

Using the CARD database, 37 ARG types were detected in 24 fecal samples (Fig. 7a, additional file 13, and additional file 14). *Bifidobacterium* had most ARGs in most samples (22/24). The 37 ARGs were resistant to nine antibiotics classes. Additionally, ARG numbers varied from 1 − 18 in each sample (Table 1). Almost all monkeys (23/24) harbored ARG resistance to rifamycin, followed by tetracyclines containing *tetW*, *MexF* and *tet* (40) detected in 18, 16 and 16 samples respectively. Macrolides, lincosamides and streptogramins (MLS), vancomycin, and multidrug-resistance (MDR) also had a high ARG abundance. However, the abundance of ARG resistant to aminoglycoside, chloramphenicol, β-lactam, and sulfonamide was low (Fig. 7b and c).

**Table 1:** Overview of antibiotic resistome in the gut microbiomes of *R. roxellanae*

Sample name	Captive/Wild	Number of ARG in the fecal samples
J38aHF	Captive	18
J31aSM	Captive	14
J32aHF	Captive	14
J34aSF	Captive	13
X22aHF	Wild	12
D13aHM	Wild	12
J36aHM	Captive	11
B01aHM	Wild	11
D15aHF	Wild	10
J33aHM	Captive	10
N07aHM	Wild	10
N10aHF	Wild	9
N09aHF	Wild	9
B03aHF	Wild	9
B06aHF	Wild	8
X21aHM	Wild	7
X23aHF	Wild	7
Q30aHM	Wild	7
D18tHF	Wild	7
D17tHF	Wild	6
Q27aHM	Wild	6
D14aHF	Wild	5
Q28aHM	Wild	3
D16tHM	Wild	1

In terms of ARG types, ARG diversity in captive monkeys was higher than in wild monkeys. ARGs resistant to aminoglycoside, chloramphenicol, β-lactam, and sulfonamide were only found in captive monkeys (Fig. 7c).

## Discussion

The snub-nosed monkey species is one of the rarest wild animals in the world. Determining biological markers is important for the protection and health evaluation of the species. The fecal microbial communities may be ideal biological marker candidates because: (1) fecal samples can be non-invasively collected at any time; (2) the fecal microbiome represents the gut microbiome and varies in accordance with intrinsic and extrinsic environmental factors; and (3) the gut microbiome maintains a relatively stable status under certain conditions. Therefore, the gut microbiome could potentially serve as a biological marker for health surveillance of *R. roxellanae* population. To demonstrate this possibility, we characterized *R. roxellanae* gut microbiomes and associated variations when impacted by physiological factors and living environments. We used 16S rRNA gene sequencing and metagenome sequencing to characterize gut microbiomes, and for the first time, diarrheal samples were studied in parallel although only two monkeys were included.

### Gut microbiome differences between wild and captive monkeys

We showed that most gut microbiomes in healthy captive or wild animals were *Firmicutes* and *Bacteroidetes* and accounted for 75 and 70% in healthy captive and wild monkeys respectively. These results were consistent with gut microbial composition in florivorous primates reported previously [45, 46]. In addition, our study also revealed that wild monkeys exhibited higher bacterial diversity than captive monkeys. However, this is inconsistent with the previous similar study about the snub-nosed monkeys [5]. It would be partially attributed to the different sampling seasons between these two researches. Our samples were collected in July when the wild monkeys have enough and rich food to eat in the forest. Besides, these wild animals were also fed with the same food as the captive animals during the feeding time. Furthermore, it would be also possible that the captive animals in this previous report might have more varied food than the wild cohorts during the research period.

On the other hand, the wild monkeys had a lower *Bacteroidetes/Firmicutes* ratio (0.7) when compared with captive healthy monkeys (1.6). This is supported by previous reports that demonstrated a lower *Bacteroidetes/Firmicutes* ratio was associated with increased energy harvest from food [47, 48]. Consistent with this, our wild monkeys had a higher abundance of *Turicibacter*, *Lachnoclostridium*, *Dialister*, *Pseudoramibacter*, and *Flavonifactor*, which were related to food fermentation to short-chain fatty acids (SCFAs) [49–51]. Moreover, the high abundance of *Firmicutes* and *Fibrobacteres* in bacterial phyla profiles and *Clostridium* and *Ruminococcus* in bacterial genus profiles in wild monkeys showed their fecal microbiomes had a stronger ability to ferment complex polysaccharides when compared with their captive counterparts. However, captive healthy monkeys possessed a high abundance of *Provotella* and *Bacteroides,* which metabolized individual sugars, AAs, plant glycans, and small peptides for growth [52, 53]. In agreement with our finding, an increased abundance of *Provotella* and *Bacteroides* was previously observed in captive colobines [54].

As microbial metabolism converts many dietary molecules to nutrients, which are absorbed and used by hosts, we examined ECs and found that fecal microbiomes in wild monkeys fermented complex dietary plant polysaccharides to generate acetate. These fecal microbiomes of the wild monkeys had a relatively high abundance of CBM44, CBM30 and CBM5, while a high abundance of CBM27 and CBM20 was observed in the fecal microbiomes of captive healthy monkeys, which might be generated by different diet compositions. Furthermore, fecal microbiomes in wild monkeys tended to metabolize pyruvate to produce acetate, while in contrast, fecal microbiomes in captive healthy monkeys tended to use pyruvate, oxaloacetate, and oxoglutarate to produce AAs. When considering important functions in host-bacterial interactions, including complex polysaccharide degradation and SCFA synthesis [55], these differential ECs and CBMs indicated that different living environments, including diet and movement limitations, may have shifted gut microbiome toward digesting different diets and deriving energy harvest from foods to meet different energy requirements.

Taken together, a higher *Bacteroidetes/Firmicutes* ratio in captive healthy monkeys reflected a change in diet and shift of metabolism pattern compared with the wild monkeys.

### Differences between diarrheal and healthy monkeys

Previous studies revealed that diarrheal monkeys had less *Bacteroidetes* and more *Firmicutes* abundance [5]. Similar changes were observed in diarrheal *R. roxellanae*. The notion that diarrhea only exists in captive monkeys (additional file 1) prompted us to investigate diarrheal causes in these monkeys. When compared with wild monkeys, captive monkeys had lower bacterial diversity (Fig. 1a) and a limited ability to synthesize SCFAs, which was reported to typically protect animals against inflammatory bowel disease [56, 57]. We further analyzed bacterial levels between captive healthy and diarrheal monkeys. *Escherichia coli* had a higher abundance in captive diarrheal monkeys (Fig. 6c). We previously identified an atypical *enteropathogenic E. coli 098* from the monkeys with diarrhea [2]. Therefore, further studies need to reveal the association between diarrhea and low SCFA production and pathogenic *E. coli* overgrowth in guts of diarrheal monkeys.

In our study, wild monkeys had lower *Bacteroidetes/Firmicutes* ratios and did not present any diarrheal samples (0/30), and captive monkeys presented 50% diarrheal samples (4/8). So, if the *Bacteroidetes/Firmicutes* ratio is used as a diarrheal marker, other factors significantly affecting bacterial diversity should be excluded. Moreover, captive monkeys with altered microbial communities might be more susceptible to diarrhea.

### ARG differences between wild and captive monkeys

We identified 37 ARG types in 24 fecal samples, which were resistant to nine antibiotic classes. It would be of significance to predict susceptible drugs for the monkeys’ medication when they are sick. Tetracycline resistance gene was the most abundant gene reported in pigs [58, 59], cattle [21], dogs [60] and chickens [61]. Also, some ARG such as MLS, MDR, and vancomycin were reported in underground waterways [62], rivers [63], and oceans [64]. Consistent with these observations, our study revealed that tetracycline, MLS, MDR, and vancomycin were the top drug types of ARG.

More interestingly, captive monkeys had higher ARG diversity than wild monkeys (Table 1), therefore original ARG sources are worthy of discussion. Generally speaking, monkeys free-living in the mountain experiences less environmental pollution and therefore environmental ARG sources in these locations are reduced. However, there still might be possible for ARG to circulate among the wild animals, surrounding livestock and humans. First, a large number of bacteria like *Escherichia, Streptococcus,* and *Enterococcus* are commonly associated with broad-spectrum ARGs [65], it would be possible that these bacteria might be the main source of the ARGs in the monkey’s feces. Unique ARGs in captive monkeys were: *CatD*, *cfxA3*, *acrD*, *novA*, *Sul2*, and *acrF*. Of these, *CatD* was associated with *Clostridium difficile*, which is a significant enteric pathogen of humans in hospital-acquired infections and livestock. Moreover, *C. difficile* transmission from humans to animals through food and the environment was previously reported [59]. Further work is needed to explain the source of enriched ARGs in these monkeys.

## Conclusion

Our study generated the following conclusions: (1) *Firmicutes* and *Bacteroidetes* were the most dominant bacteria in the gut microbiome of *R. roxellanae*; (2) gut microbiome variations were primarily determined by raising patterns (wild vs. captive) rather than sex or age; (3) a higher bacterial diversity and a lower *Bacteroidetes/Firmicutes* ratio were potential biological markers for monkey health; and (4) potential ARG transmission between the monkeys, surrounding livestock and humans should be carefully considered. Overall, humans must be mindful of the harmful effects on gut microbiome alterations resulting from captive breeding approaches and dietary niches in order to protect endangered animals.

## Supplementary Information

**Additional file 1.** Phenotype information of all monkeys.

**Additional file 2.**  Total OTUs before and after reads normalized based on a standard of sequence number corresponding to the sample with the least sequences.

**Additional file 3.** Unweighted Unifrac distance from Amplicon sequencing.

**Additional file 4.** Weighted Unifrac distance from Amplicon sequencing.

**Additional file 5.** Data Production of 24 samples.

**Additional file 6.** Relative abundance profile at genus level.

**Additional file 7.** The differential genera between captive health monkeys and wild monkeys measured by MetaStats.

**Additional file 8.** Gene abundance of each sample annoated against KEGG, eggNOG and CAZy.

**Additional file 9.** Relative abundance profile at KEGG EC level.

**Additional file 10.** Detailed information of differential KEGG ECs.

**Additional file 11.** Relative abundance profile at CAZy family level.

**Additional file 12.** Detailed information of differential CAZy family.

**Additional file 13.** Overview of the antibiotic resistome in the gut of R.roxellanae.

**Additional file 14.** The annotation results against CARD database.

**Additional file 15:** **FigureS1.** PCoA based on the Unweighted UniFrac distance from the genera profile. a,the PCoA analysis of age effect on the gut microbiomeal communities of wildfemale monkeys affected age. B, the PCoA analysis of sex effect on the gutmicrobiomeal communities of wild adult monkeys affected by sex. **FigureS2.** The overview map of metabolic pathways between wild and captive healthymonkeys. The bold blue lines represented the shared metabolic pathways betweenwild and captive healthy monkeys. The bold green lines represented uniquemetabolic pathways of captive healthy monkeys unique metabolic pathways. Thebold red lines represented unique metabolic pathways of wild monkeys uniquemetabolic pathways. **FigureS3.** Functional composition of the gut metagenome in the eggNOG database. Thebar length iwas scaled with the number of genes. **FigureS4.** The distribution of Enzyme Classes between different samples. In thecenter, the Bray-Curtis distance cluster tree was calculated from the relativeabundance profile. C: captive monkeys; W: wild monkeys.  

## Data Availability

The datasets presented in this study can be found in online repositories. The names of the repository and accession number(s) can be found below: EMBL-EBI, PRJEB14698.
