# Supplementary material for: Gut microbiome variations in Rhinopithecus roxellanae caused by changes in the environment
Source: BMC Genomics. 2023 Feb 3;24:62. doi: 10.1186/s12864-023-09142-6 (PMC9896789; doi:10.1186/s12864-023-09142-6)
Supplement: Supplementary file 15 — Additional file 15: FigureS1. PCoA based on the Unweighted UniFrac distance from the genera profile. a,the PCoA analysis of age effect on the gut microbiomeal communities of wildfemale monkeys affected age. B, the PCoA analysis of sex effect on the gutmicrobiomeal communities of wild adult monkeys affected by sex. FigureS2. The overview map of metabolic pathways between wild and captive healthymonkeys. The bold blue lines represented the shared metabolic pathways betweenwild and captive healthy monkeys. The bold green lines represented uniquemetabolic pathways of captive healthy monkeys unique metabolic pathways. Thebold red lines represented unique metabolic pathways of wild monkeys uniquemetabolic pathways. FigureS3. Functional composition of the gut metagenome in the eggNOG database. Thebar length iwas scaled with the number of genes. FigureS4. The distribution of Enzyme Classes between different samples. In thecenter, the Bray-Curtis distance cluster tree was calculated from the relativeabundance profile. C: captive monkeys; W: wild monkeys. [file 12864_2023_9142_MOESM15_ESM.docx]

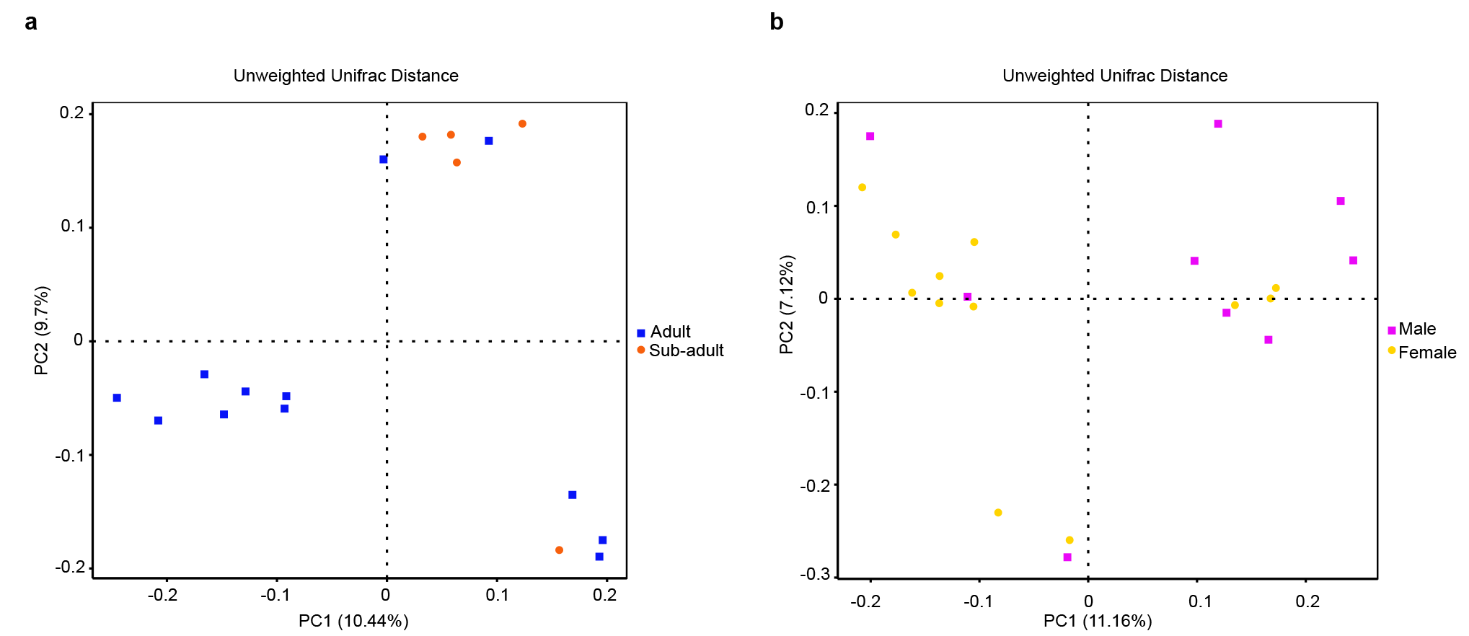


Figure S1. PCoA based on the Unweighted UniFrac distance from the genera profile. a, the PCoA analysis of age effect on the gut microbiomeal communities of wild female monkeys affected age. B, the PCoA analysis of sex effect on the gut microbiomeal communities of wild adult monkeys affected by sex.


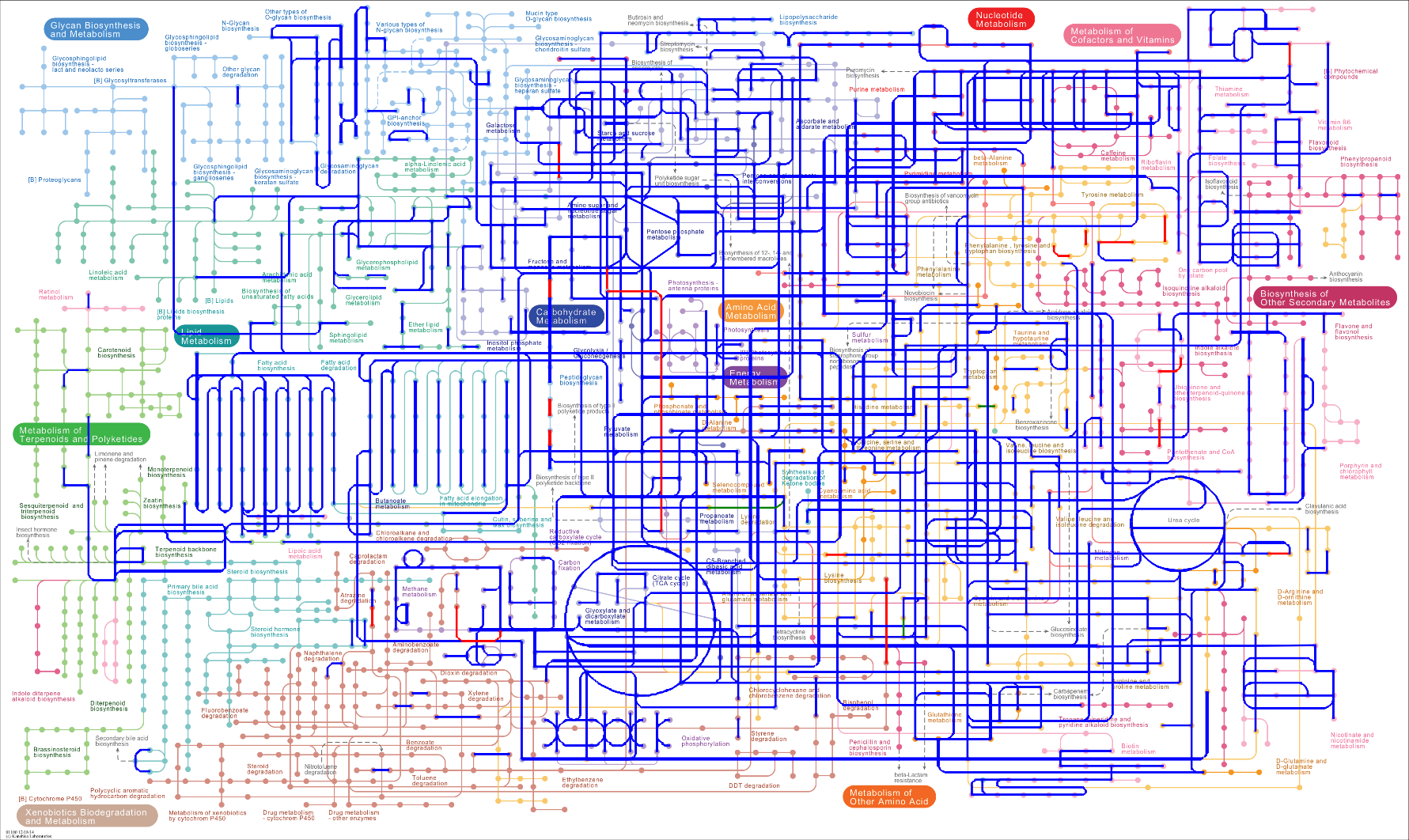


Figure S2. The overview map of metabolic pathways between wild and captive healthy monkeys. The bold blue lines represented the shared metabolic pathways between wild and captive healthy monkeys. The bold green lines represented unique metabolic pathways of captive healthy monkeys unique metabolic pathways. The bold red lines represented unique metabolic pathways of wild monkeys unique metabolic pathways.


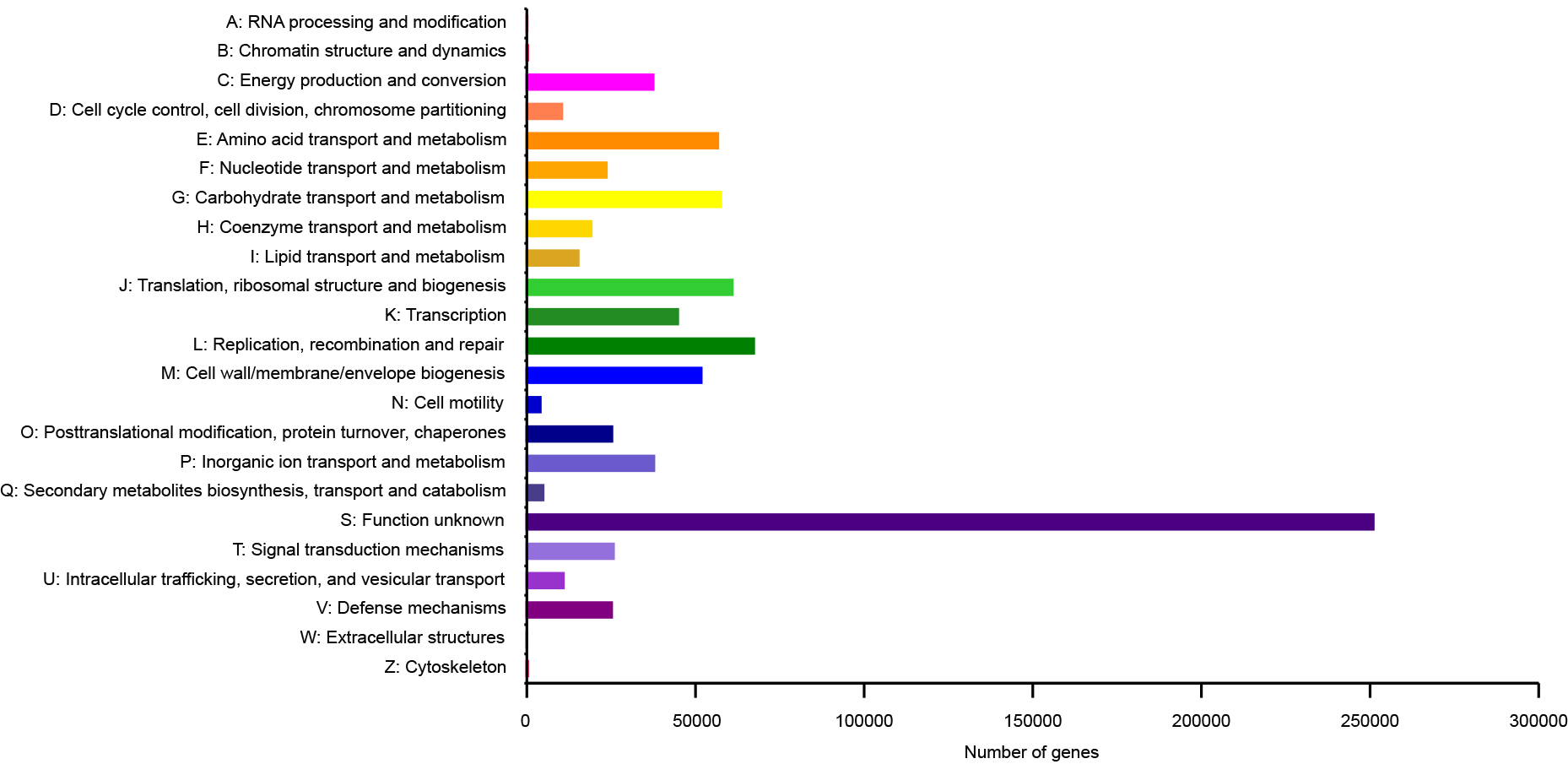


Figure S3. Functional composition of the gut metagenome in the eggNOG database. The bar length iwas scaled with the number of genes.


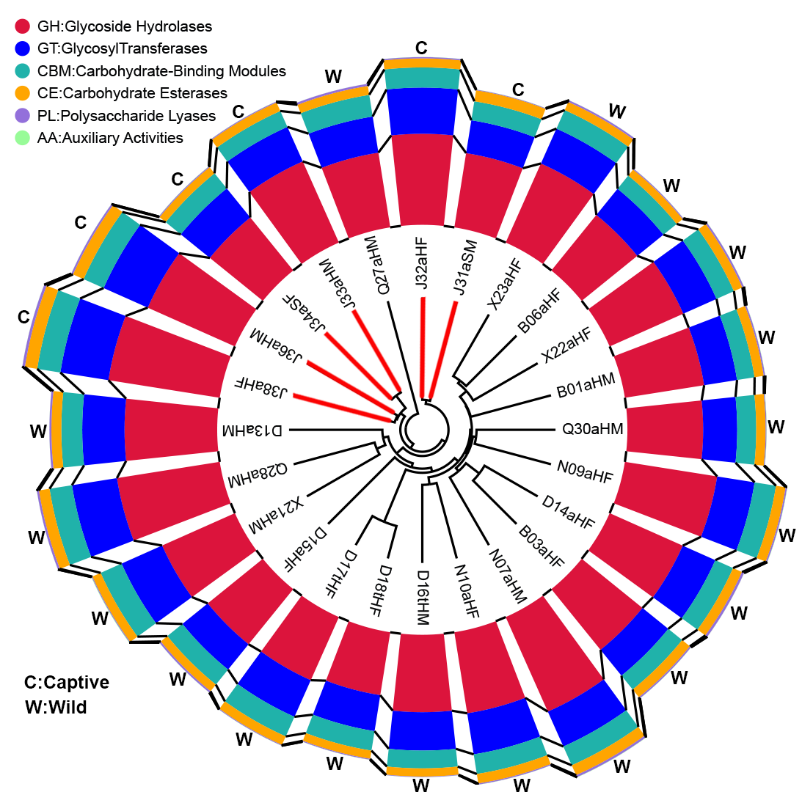


Figure S4. The distribution of Enzyme Classes between different samples. In the center, the Bray-Curtis distance cluster tree was calculated from the relative abundance profile. C: captive monkeys; W: wild monkeys.
